# Effects of a Rehabilitation Program on Perceived Environmental Barriers in Older Patients Recovering from Hip Fracture: A Randomized Controlled Trial

**DOI:** 10.1155/2013/769645

**Published:** 2013-08-06

**Authors:** Erja Portegijs, Merja Rantakokko, Johanna Edgren, Anu Salpakoski, Ari Heinonen, Marja Arkela, Mauri Kallinen, Taina Rantanen, Sarianna Sipilä

**Affiliations:** ^1^Gerontology Research Center and Department of Health Sciences, University of Jyväskylä, P.O. Box 35 (viv), 40014 Jyväskylä, Finland; ^2^Department of Health Sciences, University of Jyväskylä, P.O. Box 35, 40014 Jyväskylä, Finland; ^3^Department of Physiotherapy, Central Finland Central Hospital, Keskussairaalantie 19, 40620 Jyväskylä, Finland; ^4^Department of Physical Medicine and Rehabilitation, Central Finland Central Hospital, Keskussairaalantie 19, 40620 Jyväskylä, Finland; ^5^Department of Medical Rehabilitation, Oulu University Hospital, P.O. Box 21, 90029 Oulu, Finland

## Abstract

*Objectives*. To study effects of a one-year multicomponent intervention on perceived environmental barriers in hip fracture patients. *Design*. Randomized controlled trial of a 12-month home-based rehabilitation aiming to improve mobility and function (ISRCTN53680197); secondary analyses. *Subjects*. Community-dwelling hip fracture patients on average 70 days after trauma (*n* = 81). *Methods*. Assessments at baseline, 3, 6, and 12 months later included perceived entrance-related barriers (e.g., indoor/outdoor stairs, lighting, floor surfaces, and storage for mobility devices) and perceived barriers in the outdoor environment (poor street condition, hilly terrain, long-distances, and lack of resting places). Sum scores for entrance-related and outdoor barriers were analyzed using general estimating equation models. *Results*. At baseline, 48% and 37% of the patients perceived at least one entrance-related barrier, and 62% and 60% perceived at least one outdoor barrier in the intervention and control group, respectively. Over time, (*P* = 0.003) the number of entrance-related barriers decreased in both groups (group *P* = 0.395; interaction *P* = 0.571). For outdoor barriers, time (*P* = 0.199), group (*P* = 0.911), and interaction effect (*P* = 0.430) were not significant. *Conclusion*. Our intervention had no additional benefit over standard care in hip fracture patients. Further study is warranted to determine whether perceived environmental barriers can be reduced by interventions targeted at the older individual. This trial is registered with ISRCTN53680197.

## 1. Introduction

Loss of the ability to move outdoors may threaten independence and quality of life of community-dwelling older people [1–5]. Outdoor mobility requires a certain level of functional capacity along with the ability to concentrate and react on environmental stimuli [6]. When environmental demands exceed the capability of a person, environmental barriers arise and cause difficulty in moving around [6–8]. Loss of function, such as what occurs following hip fracture, may exacerbate this difficulty. After hip fracture less than one-third of those survived recover their prefracture level of functioning within one year [9, 10]. Additionally, they are at high risk for disability, becoming homebound, and losing independence [11]. Outdoor mobility may be challenging and in the presence of environmental barriers even an insurmountable obstacle, leaving some people homebound.

Self-reported environmental barriers [2, 7, 12–14] reflect a person's abilities (physical and mental) and resources (e.g., availability of devices or personal aid) as well as characteristics of the physical environment that a person uses (e.g., heavy doors, stairs, slopes, and uneven surface). Most studies have been performed in larger city areas. Barriers are environment specific. In Finland the environment may include hills, slopes, and large-spaced areas. In addition, the climate includes cold winters and warm summers. Cold temperature per se may not prevent Finnish people to go outdoors; however, slippery surface makes moving outdoors more challenging occasionally. Environmental characteristics [12, 15, 16] and the weather [17, 18] may thus affect perceived environmental barriers.

In older people following hip fracture, physical functioning, for example, mobility, balance, and strength, may improve by rehabilitation including progressive resistance training [19–21]. Considering the relationship between physical function and perceived barriers in the outdoor environment [7, 12], improvements in functioning may also affect the perception of environmental barriers. Improvements in functioning may remove previously reported environmental barriers by that person, without a change in the environment itself. On the other hand, improved functioning may also increase the amount of outdoor physical activity [22]. This may increase exposure to the outdoor environment, thus increasing the awareness of environmental barriers [23]. In addition, awareness may be increased by interventions targeting the indoor and outdoor environment [24, 25].

To our knowledge, studies of intervention effects on perceived environmental barriers in and surrounding a person's home are not available for clinical groups, such as hip fracture patients. Our aim was to study the effects of a multicomponent intervention on perceived barriers in the environment. The intervention aimed to promote recovery in mobility and function in patients following hip fracture. We hypothesized that improvements in functioning that were expected to be larger in the intervention group would remove perceived barriers in the entrance-related and outdoor environment a person uses.

## 2. Methods

### 2.1. Study Design and Participants

Secondary analyses of a randomized controlled trial of multicomponent home-based rehabilitation in community-dwelling patients following hip fracture (ISRCTN53680197). Study methods have been described before [26]. Data were collected in a research center from 44 to 239 days following the fracture (baseline) and three, six, and twelve months later. All assessors were blinded to study group.

**Figure 1:**
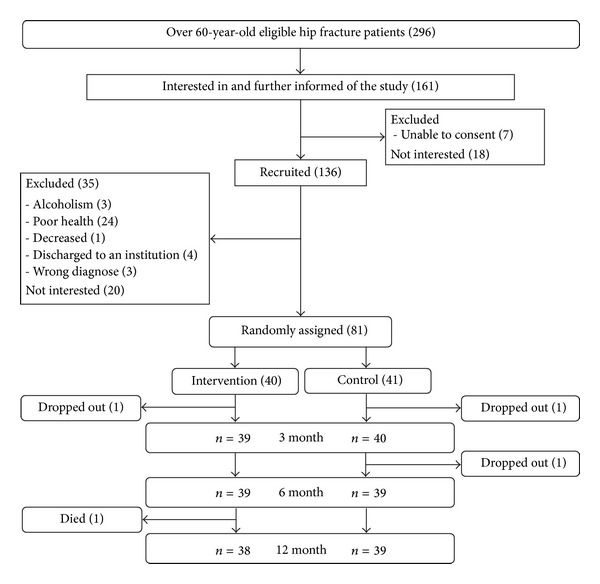
Flow chart of the study.

Patient records at the Central Hospital of the catchment area were reviewed to recruit all community-dwelling people over 60 years old, operated for femoral neck or trochanteric fracture from 1.3.2008 to 31.12.2010 (*N* = 296; flow chart in Figure 1). Those willing to participate were met during the inpatient period at the health care center to ensure suitability (*N* = 136). Patients living in an institution or being bedridden at the time of fracture, suffering from severe memory problems (Mini Mental State Examination < 18), alcoholism, severe cardiovascular, pulmonary or progressive (i.e., neoplasm, ALS) disease, para- or tetraplegic, or severe depression (Beck Depression Inventory > 29) were excluded. After baseline assessments, eligible patients were randomly allocated to the intervention (*N* = 40) and control (*N* = 41) groups using a computer-generated list. Blocks of 10 by gender and operation type (internal fixation/arthroplasty) were used. The study power was calculated for mobility limitation [26]. The study protocol was approved by the ethical committee of the local Health Care District and complies with the Declaration of Helsinki. All participants gave their written informed consent prior to the assessments. 

### 2.2. Assessments

A physician and research nurse performed a clinical examination. Details of the fracture, its repair (osteosynthesis *n* = 38, total or hemiarthroplasty *n* = 43), and chronic conditions were confirmed according to a questionnaire and medical records. *The number of chronic diseases* present for at least three months was calculated as an indicator of comorbidity. *Time since fracture* was defined as the number of days between the date of fracture and baseline assessment. 

Barriers to mobility in the outdoor and entrance environments of the home were examined as perceived by the participants using standardized questionnaires. The questions on environmental barriers were developed by an expert panel for an earlier study (Screening and Counseling for Physical Activity and Mobility project) [27]. *Perceived entrance-related barriers *(PEB) included indoor and outdoor stairs, elevator, steps, doors, lighting, floor surfaces, and lack of suitable storage for mobility devices. Participants were asked to rate whether each item hindered their mobility (0: no, 1: yes). *Perceived outdoor barriers *(POB) included streets in poor condition, terrain with slopes, long distances to services, and lack of resting places (benches). Participants were asked to rate whether each item reduced their possibility to move independently (0: no, 1: yes). Sum scores were calculated for PEB (range 0–8) and POB (range 0–4). The presence of terrain- or distance-related barriers assessed with the same scale doubled the risk for developing outdoor mobility limitation over a 3.5-year period in a previous study [7]. In addition, the presence of these barriers was associated with poorer quality of life [2].

Participants were asked to rate their *perceived difficulty to walk indoors* without a walking aid and *perceived difficulty to walk outdoors* (with or without walking aid). Three categories were created: (1) no difficulty, (2) some difficulty, and (3) major difficulty or unable (with or without help from another person). The present *level of physical activity* was assessed with a self-report scale by Grimby [28] with slight modifications. Three categories were created: (1) mostly sitting or resting, (2) light physical activity, for example, light house hold tasks, and (3) moderate physical activity, for example, walking longer distance, domestic work, and/or more strenuous activity. Self-reported *housing-related features *at baseline included characteristics of the house (block of apartments, attached house, and semiattached/separate family home) and neighborhood (urban, suburban, and rural). *Vision acuity *at 5 m distance was measured in the research center with and without participants' own spectacles with the illuminated Landolt ring chart (Oculus 4512). We used the best (highest) score of both eyes together. The *average temperature* of the month in which the respective assessment took place was derived from average monthly temperature data, collected in the years 1981–2010 daily at 12 o'clock at a local weather station in Jyväskylä [29].

### 2.3. Intervention

Standard care [26] is typically comprised of a written home exercise program (without additional resistance or updates) from the hospital or health care center. A referral to physiotherapy was occasionally prescribed (intervention group *n* = 5; control group *n* = 7). The control group received standard care only. The intervention group received an individually tailored year-long rehabilitation intervention [26] in addition to standard care. The intervention included five to seven home visits by an experienced physiotherapist and comprised the following components: (1) a checklist-based *evaluation on modifiable environmental hazards* known to increase fall risk [30]: if necessary, participants were advised on environmental factors, such as furniture arrangement, removal of mats, or addition of hand grips. We did not check adherence to the advice given; (2) *guidance for safe walking* including readjustment of walking aids and information on shoes and antislip shoe devices for icy conditions; (3) *pain assessment* and discussions on pain relief strategies; (4) *progressive home exercise program* including strengthening exercises for the lower limbs, balance training in standing position, functional exercises (including walking, reaching, turning, and getting up from a chair), and stretching exercises: strengthening/stretching exercises (3 times a week) and balance/functional exercises (2-3 times a week) were recommended to be performed on consecutive days. Each exercise session lasted approximately 30 minutes. The exercise program was updated 4-5 times with a more intensive and demanding protocol. The progression for the strengthening exercises was increased with resistance bands. Participants kept daily exercise diary, on which they marked completion of each exercise session and the score on the Borg Scale; (5) *individual motivational physical activity counseling* session including a personal physical activity plan. The physiotherapist supported the program adherence and the behavior change through three phone contacts and two face-to-face sessions with 1-2 months interval, starting at three months from baseline. 

### 2.4. Statistical Analyses

Normality of data distribution was tested with Kolmogorov-Smirnov tests. Within-group changes were tested using Friedman's two-way analysis of variance rank test. Intervention effects were analyzed using General Estimate Equation (GEE) modeling for count variables using the intention-to-treat principle. Crude (Model 1) and adjusted models were run, including (2) demographic (age, gender) and hip-fracture-related variables (time since fracture, number of chronic diseases), (3) level of physical activity, (4) difficulty in indoor (PEB models) or outdoor (POB models) walking, (5) visual acuity, (6) average outdoor temperature, or (7) housing-related features. The analyses were repeated for subgroups of those with and without baseline PEB or POB, respectively. Due to small sample size, crude GEE models were computed only for participants with baseline PEB ≥ 1 or POB ≥ 1, respectively. Statistical analyses were performed using PASW statistics 18 (SPPS software). Statistical significance was set at *P* < 0.05.

## 3. Results

During the course of the study, two participants were lost to follow-up in the intervention and two in the control group (Figure 1). Adherence rates (ratio sessions realized/planned) were 98% for the first face-to-face counseling session, and 83–90% for the following counseling sessions. Adherence to the exercise program (ratio exercises performed/planned) was 61% during the first and 40% during the final six months (overall 54%). The intervention was well tolerated, and no intervention related adverse events occurred. A physician suspended nine participants of the intervention group, for medical reasons (revision operation, new fractures, cardio-vascular and pulmonary events, and infections); two were suspended temporarily and two permanently during the first 6 months, and five participants were permanently suspended during the final six months.

Participants were on average 80 years old, 70 days had passed since the hip fracture, and they had on average three chronic diseases [26]. The median visual acuity was 0.7 (range 0.1–4.0) and 0.6 (0.1–1.0) in the intervention and control group, respectively. About one-third of the participants reported to have little physical activity (mostly sitting), about 60% engaged in light physical activity only (Table 1). 

### 3.1. Perceived Environmental Barriers

**Figure 2:**
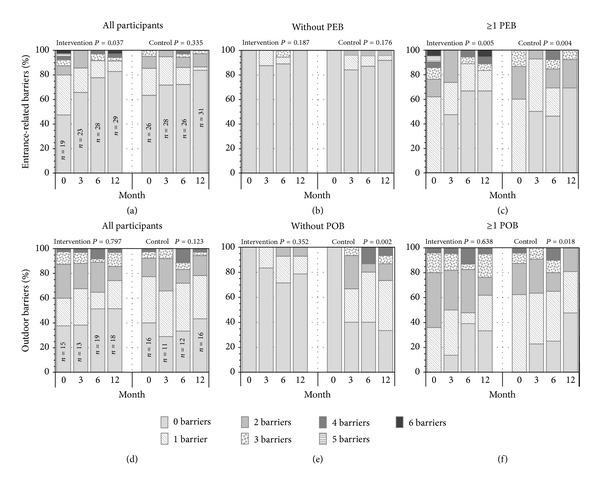
Proportions of participants in the intervention and control group according to the number of entrance-related (PEB) or outdoor (POB) barriers, for all participants and in subgroups of those with and without baseline PEB or POB, respectively. The within-group change over time was tested with Friedman two-way analysis of variance rank tests.

At baseline, 48% and 37% of the participants reported at least one PEB, and 62% and 60% reported at least one POB in the intervention and control groups, respectively (Figure 2). Within-group changes over time showed a significant decrease in number of PEB only in the intervention group (Friedman *P* = 0.037). In participants with baseline PEB ≥ 1, this decrease was found in both groups (*P* ≤ 0.004). In those without baseline PEB, few persons perceived new barriers at follow-up assessments (*P* ≥ 0.176). In the main analyses, no significant change over time in POB occurred in either group (*P* ≥ 0.123). In the subanalyses, the number of barriers significantly decreased in control group participants with baseline POB ≥ 1 (*P* = 0.018) and increased in the control group participants without baseline POB (*P* = 0.002).

### 3.2. Multivariate Models

Table 2 shows crude and adjusted GEE models for PEB and POB. PEB decreased significantly over time (*P* = 0.003) in both groups. Accounting for demographic factors, vision, and housing-related features did not markedly change the results. However, reduced difficulty to walk indoors and increased physical activity partly accounted for the decrease in PEB rendering “time” nonsignificantly (*P* ≥ 0.211). In subanalyses, PEB decreased over time (*P* < 0.001) in both groups (group *P* = 0.748; interaction *P* = 0.400) in those with baseline PEB ≥ 1.

For POB, the effects of group, time, and interaction were not significant (*P* ≥ 0.189; Table 2). Accounting for difficulty to walk outdoors resulted in a significant decrease over time (*P* = 0.049) in both groups. Adjusting for level of physical activity resulted in a near significant effect of time (*P* = 0.055). Accounting for other variables did not markedly change the results. In subanalyses, POB decreased over time (*P* = 0.007) in both groups (group *P* = 0.100; interaction *P* = 0.239) in those with baseline POB ≥ 1.

## 4. Discussion

The one-year multicomponent rehabilitation program in older people starting on average three months after hip fracture did not have added value over standard care in terms of larger reductions in perceived barriers related to the entrance or outdoor environment. Yet, overall the number of perceived barriers decreased in these relatively well-functioning patients. The reduction in number of PEB and POB over time was partly explained by reduced difficulty in walking and increased level of physical activity in the months following the fracture. Other factors, including demographics, housing-related features, visual acuity, and outdoor temperature, did not affect the change in PEB or POB. More than half of the participants reported no PEB and about 40% no POB at baseline. In this group, improvements in functioning would thus not lead to a reduction in the number of barriers. Nevertheless, subanalyses in participants reporting at least 1 barrier at baseline did not change the results markedly. 

Our hypothesis that an intervention might change perceptions of environmental barriers in patients following hip fracture was based on the competence-press model [6] or person-environment fit model [8]. There is a continuous interaction between a person, his/her competencies, and the environment, which poses a set of demands. Thus, when the functional capacity of a person decreases drastically due to a hip fracture, the environment poses more demands on the person, which will affect the perception of environmental barriers. Concurrently improvements in functioning, occurring in the recovery from hip fracture, may reduce the number of perceived environmental barriers. Since improvements in function were expected to be larger in the intervention group, a significant intervention effect was expected. 

Intervention effects on perceived environmental barriers have hardly been studied before. Previous studies mainly comprise of cross-sectional data, in which associations between perceived environmental barriers, functional capacity, and characteristics of the environment have been shown [12, 15, 16, 31]. These studies commonly showed that differences in the environment account for many health-related behaviors and health indicators [5, 31, 32]. It is currently unknown whether perceived environmental barriers can be reduced in older people by interventions targeting the individual. Targeting the environment of an individual may reduce poor functional outcomes [32]. Our intervention mainly targeted mobility and balance function [26]. Also it included an evaluation of modifiable environmental hazards in or directly outside of the home, mainly aiming at falls reduction. A study by Di Monaco et al. [25] showed that a single home visit reduced the number of falls after hip fracture, especially in those adhering to the advice. Advice on modifiable factors may raise awareness of environmental barriers [24, 25]. However, in our study many participants had already made modifications to their homes (e.g., hand grips on walls, removal of mats) prior to the visit of our physiotherapist. Unfortunately, we did not check adherence to the advice given.

Our study did not show added value of the intervention over standard care; over time the number of PEB decreased on average in both groups. After adjustment for level of physical activity, a proxy for the amount of exposure to the outdoor environment, the number of POB decreased over time, indicating at least partial mediation by increased physical activity and decreased difficulty to walk outdoors. Increasing or maintaining physically active, thus using the affected muscles, aids the rehabilitation process following hip fracture [33]. Consequently, rehabilitation effects may be more sustainable in the absence of perceived barriers in the home environment, yet this needs to be confirmed in future studies. 

To our knowledge, this is the first study on perceived environmental barriers in older people following a hip fracture. Studying perceived environmental barriers in clinical groups is relevant, since they are at high risk for adverse events, such as disability, becoming homebound, and loss of independence. Efforts to prevent these outcomes may help the individual, in terms of quality of life, as well as society, in terms of cost reduction. However, recent hip fracture patients may have difficulty to correctly identify barriers, especially outside the home. At baseline, some participants may not have moved outdoors independently since fracture. They may have rated barriers based on their experiences prior to the fracture, being too optimistic. A few months later, when they started to move outdoors and thus exposure to environmental barriers increased, they perceived additional barriers, which confounded the relationship. This was visible especially in the control group where 9/15 participants developed new POB within three months (versus intervention group 2/12). Further study is needed to determine factors underlying the rating of perceived environmental barriers by hip fracture patients and other clinical populations in different phases of the recovery process. 

The measure used to assess perceived environmental barriers has been used in previous studies. Terrain- and distance-related outdoor barriers predicted the development of mobility limitation [7]. In addition, outdoor barriers have been associated with quality of life [2]. Previously our measure has not been used to assess changes in time. In the current study changes in perceived barriers over time occurred conform our preexisting hypothesis. Thus the measure seems responsive to change. However, the measure may not be sensitive enough to detect subtle changes or differences between groups. Partly this may also be due to the fact that the actual environment of our study participants did not change due to the intervention. Further, the measure used had a ceiling effect (about half of the participants reporting no barriers). In these participants, there was no potential for improvement by intervention. Thus the changes over time were more evident when those with preexisting barriers were analyzed separately.

Generalization of the study results should happen with caution. Our patients were relatively well functioning when compared to the general population of hip fracture patients due to our inclusion criteria (community dwelling and no serious cognitive impairment). In addition, the sample size was rather small for the presented outcome as study power was calculated for mobility limitation [26]. Compared to similar rehabilitation programs, the adherence to the home-based exercises was comparable [34, 35], and adherence to the physical activity counseling sessions was excellent. Our participants were living in a large variety of environments, as the recruitment area included urban, suburban, and rural areas. Environmental barriers may differ according to type of housing and living area. Unfortunately, our sample size was too small to do subgroup analyses. Adjustment for housing-related features, however, did not materially change the relationships. Unfortunately, we do not know about the environmental barriers participants perceived prior to the hip fracture. Thus it remains unclear whether the number of perceived environmental barriers ever returned to their prefracture level. 

## 5. Conclusion

In patients following hip fracture, the combination of function loss and barriers in the environment may pose an insurmountable obstacle to move outdoors or maintain independence. Our multidisciplinary intervention had no additional benefit over standard care in terms of larger reductions in perceived environmental barriers in this group of rather well-functioning individuals following hip fracture. Yet, overall the number of perceived barriers related to the entrance and outdoor environment decreased within the one-year followup. Further study is warranted to determine whether perceived environmental barriers can be reduced by intervention targeted at the older individual and whether more specific target groups should be selected for intervention. 

## Figures and Tables

**Table 1:** Participants characteristics in the intervention (*N* = 40) and control groups (*N* = 41) at baseline.

	Intervention group		Control group	
	*N*	%	*N*	%
*Walking aid for inside *				
Yes	27	67.5	25	61.0
*Difficulty walking indoors (without walking aid) *				
Without	13	32.5	17	41.5
Some	7	17.5	12	29.3
Major difficulty/unable	20	50	12	29.3
*Walking difficulty outdoors (with/without walking aid) *				
Without	4	10	8	19.5
Some	18	45	20	48.8
Major difficulty/unable	18	45	13	31.7
*Level of physical activity *				
Mostly sitting	15	37.5	11	27.5
Light physical activity	23	57.5	25	62.5
Moderate physical activity	2	5	4	10
*Home *				
Block apartments	18	45	16	39
Attached house	10	25	7	17.1
Semiattached/separate house	12	30	18	43.9
*Neighborhood *				
Urban	18	45.0	16	39.0
Suburban	13	32.5	15	36.6
Rural	9	22.5	10	24.4

**Table 2:** The independent and interaction effects of time and group for perceived entrance-related barriers and perceived outdoor barriers derived from the GEE models.

	Perceived entrance-related barriers			Perceived outdoor barriers		
	Time	Group	Interaction	Time	Group	Interaction
Model 1	0.003	0.395	0.517	0.189	0.911	0.430
Model 2	0.001	0.303	0.499	0.185	0.793	0.433
Model 3	0.230	0.254	0.396	0.055	0.674	0.548
Model 4	0.211^a^	0.665^a^	0.467^a^	0.049^b^	0.439^b^	0.528^b^
Model 5	0.003	0.315	0.461	0.254	0.456	0.501
Model 6	0.001	0.529	0.493	0.096	0.993	0.500
Model 7	0.003	0.459	0.475	0.164	0.735	0.322

Model 1 is crude model, Model 2 adjusted for demographic (age, gender) and hip-fracture-related variables (time since fracture, number of chronic diseases), Model 3 adjusted for level of physical activity, Model 4 adjusted for ^a^difficulty to walk indoors or ^b^difficulty to walk outdoors, Model 5 adjusted for visual acuity, Model 6 adjusted for average outdoor temperature, and Model 7 adjusted for housing-related features.
